# (Bipyridine-κ^2^ *N*,*N*′)chlorido[*N*-(2-hydroxy­ethyl)-*N*-isopropyl­dithio­carbamato-κ^2^ *S*,*S*′]zinc(II)

**DOI:** 10.1107/S1600536812027626

**Published:** 2012-06-23

**Authors:** Fatin Allia Mohamad, Ibrahim Baba, Mohamed Ibrahim Mohamed Tahir, Edward R. T. Tiekink

**Affiliations:** a School of Chemical Sciences and Food Technology, Faculty of Science and Technology, Universiti Kebangsaan Malaysia, 43600 Bangi, Malaysia; b Department of Chemistry, Universiti Putra Malaysia, 43400 Serdang, Malaysia; c Department of Chemistry, University of Malaya, 50603 Kuala Lumpur, Malaysia

## Abstract

The Zn^II^ atom in the title compound, [Zn(C_6_H_12_NOS_2_)Cl(C_10_H_8_N_2_)], is coordinated by a chelating *N*-2-hy­droxy­ethyl-*N*-isopropyl­dithio­carbamate ligand, a 2,2′-bipyridine ligand and a Cl atom. The resulting ClN_2_S_2_ donor set defines a distorted square-pyramidal coordination geometry. Helical supra­molecular chains sustained by O—H⋯S hydrogen bonds and propagating along the *b* axis feature in the crystal packing. A three-dimensional architecture is stabilized by C—H⋯O, C—H⋯S and C—H⋯Cl inter­actions.

## Related literature

For crystal engineering studies on zinc complexes with fuctionalized dithio­carbamate ligands, see: Benson *et al.* (2007 ▶); Poplaukhin & Tiekink (2010 ▶). For the distinction between square-pyramidal and trigonal-bipyramidal geometries, see: Addison *et al.* (1984 ▶).
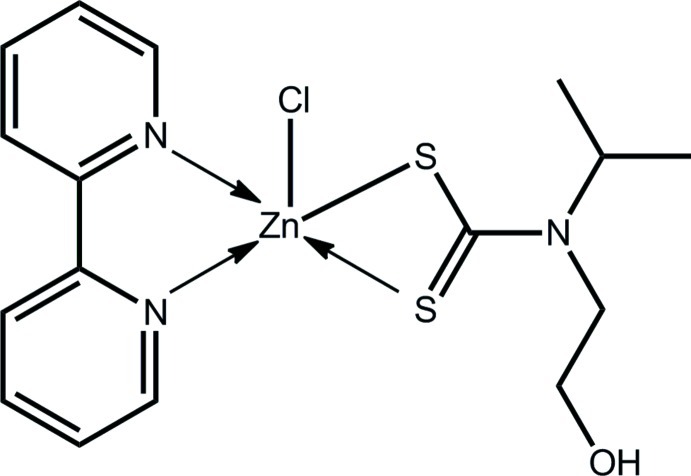


## Experimental

### 

#### Crystal data

[Zn(C_6_H_12_NOS_2_)Cl(C_10_H_8_N_2_)]
*M* *_r_* = 435.29
Monoclinic, 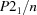

*a* = 14.5008 (10) Å
*b* = 8.6216 (4) Å
*c* = 15.9905 (9) Å
β = 114.423 (7)°
*V* = 1820.25 (18) Å^3^
*Z* = 4
Mo *K*α radiation
μ = 1.73 mm^−1^
*T* = 100 K
0.35 × 0.20 × 0.12 mm

#### Data collection

Oxford Diffraction Xcaliber Eos Gemini diffractometer
Absorption correction: multi-scan (*CrysAlis PRO*; Agilent, 2011 ▶) *T*_min_ = 0.882, *T*_max_ = 1.000
11927 measured reflections
4061 independent reflections
3603 reflections with *I* > 2σ(*I*)
*R*_int_ = 0.034

#### Refinement

*R*[*F* ^2^ > 2σ(*F* ^2^)] = 0.031
*wR*(*F* ^2^) = 0.071
*S* = 1.04
4061 reflections
222 parameters
1 restraint
H atoms treated by a mixture of independent and constrained refinement
Δρ_max_ = 0.83 e Å^−3^
Δρ_min_ = −0.37 e Å^−3^

### 

Data collection: *CrysAlis PRO* (Agilent, 2011 ▶); cell refinement: *CrysAlis PRO*; data reduction: *CrysAlis PRO*; program(s) used to solve structure: *SHELXS97* (Sheldrick, 2008 ▶); program(s) used to refine structure: *SHELXL97* (Sheldrick, 2008 ▶); molecular graphics: *ORTEP-3 for Windows* (Farrugia, 1997 ▶) and *DIAMOND* (Brandenburg, 2006 ▶); software used to prepare material for publication: *publCIF* (Westrip, 2010 ▶).

## Supplementary Material

Crystal structure: contains datablock(s) global, I. DOI: 10.1107/S1600536812027626/bt5940sup1.cif

Structure factors: contains datablock(s) I. DOI: 10.1107/S1600536812027626/bt5940Isup2.hkl

Additional supplementary materials:  crystallographic information; 3D view; checkCIF report

## Figures and Tables

**Table 1:** Selected bond lengths (Å)

Zn—Cl1	2.2503 (5)
Zn—S1	2.4366 (6)
Zn—S2	2.5026 (6)
Zn—N2	2.1097 (18)
Zn—N3	2.1692 (18)
S1—C1	1.736 (2)
S2—C1	1.723 (2)

**Table 2:** Hydrogen-bond geometry (Å, °)

*D*—H⋯*A*	*D*—H	H⋯*A*	*D*⋯*A*	*D*—H⋯*A*
O1—H1*o*⋯S1^i^	0.84 (2)	2.42 (2)	3.2528 (18)	167 (2)
C6—H6c⋯O1^i^	0.98	2.47	3.438 (3)	169
C13—H13⋯S2^ii^	0.95	2.81	3.625 (2)	144
C7—H7⋯Cl1^iii^	0.95	2.79	3.579 (3)	142
C8—H8⋯Cl1^iv^	0.95	2.76	3.606 (2)	148

Symmetry codes: (i) 
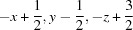
; (ii) 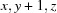; (iii) 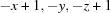; (iv) 
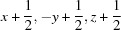
.

**Fig. 1.**
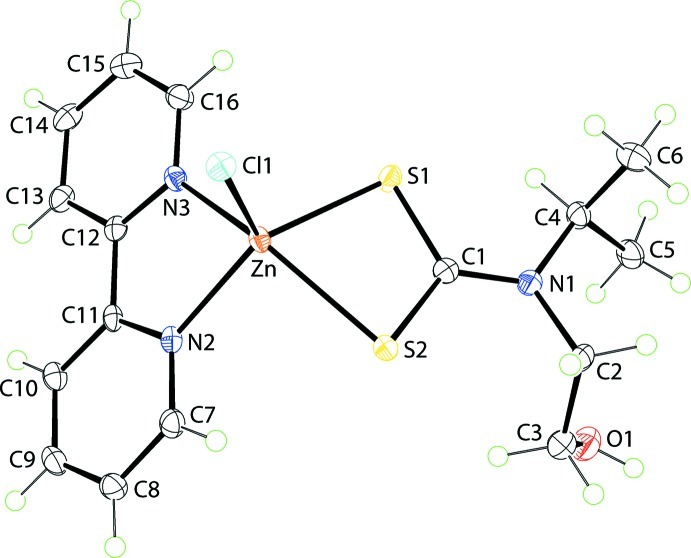
The molecular structure of (I) showing the atom-labelling scheme and displacement ellipsoids at the 50% probability level.

**Fig. 2.**
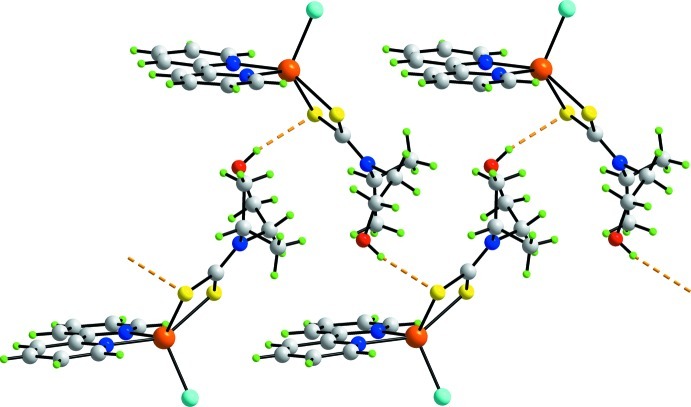
A view of the helical supramolecular chain in (I) mediated by O—H···S hydrogen bonds (orange dashed lines) along the b axis.

**Fig. 3.**
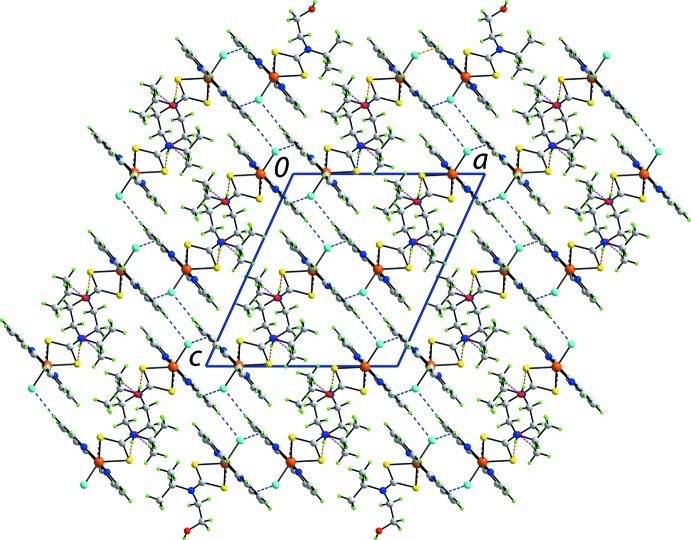
A view of the crystal packing in projection down the b axis. The O—H···S, C—H···O, C—H···S and C—H···Cl interactions are shown as orange, pink, brown and blue dashed lines, respectively.

## supplementary crystallographic information

### Comment

Introducing hydroxylethyl functionality into dithiocarbamate ligands facilitates the formation of higher dimensionality in their crystal structures (Benson *et al.*, 2007; Poplaukhin & Tiekink, 2010). As a continuation of these studies, herein, the title compound, Zn[S_2_CN(CH_2_CH_2_OH)iPr](2,2'-bipyridine)Cl, (I), is described.

The molecular structure of (I), Fig. 1, features a Zn^II^ atom coordinated by a dithiocarbamate ligand, two N atoms of the 2,2'-bipyridine ligand and a chloride. The dithiocarbamate ligand coordinates essentially in a bidentate mode, an assignment supported by the near equivalence of the C—S bond distances, Table 1. The resulting ClN_2_S_2_ donor set defines a coordination geometry intermediate between square pyramidal and trigonal bipyramidal geometry. This is quantified by the value of τ = 0.23 which compares to the τ values of 0.0 and 1.0 for ideal square pyramidal and trigonal bipyramidal geometries, respectively (Addison *et al.*, 1984).

The crystal packing of (I) features helical supramolecular chains along the *b* axis that are sustained by O—H···S interactions, Fig. 2 and Table 2. Additional stability to the chains are afforded by C—H···O and C—H···S interactions, Table 2. The chains are connected into a three-dimensional architecture by C—H···Cl interactions, Fig. 3 and Table 1.

### Experimental

This compound was prepared using the *in situ* method by the addition of carbon disulfide (0.01 mol) to an ethanolic solution of isopropylethanolamine (0.01 mol). The mixture was stirred for one hour at 277 K. Then, it was added drop-wise to a solution of zinc dichloride (0.005 mol) in ethanol (20 ml) followed by 2,2'-bipyridine (0.01 mol) in ethanol (20 ml). The mixture was stirred for another two hours at 277 K. The white precipitate was filtered, washed with cold ethanol and dried in a desiccator. Crystallization was from its ethanol:chloroform (1:2) solution held at room temperature. Yield: 65.5%. *M*.pt. 424–426 K. Elemental analysis. Found (calculated) for C_16_H_20_ClN_3_OS_2_Zn: C, 45.21 (44.70); H 3.59 (3.50); N 9.69 (9.70); S 15.12 (14.90)%. IR (KBr): ν(O—H) 3391 m; ν(C≐N) 1442 s; ν(C≐S) 944 s; ν(Zn—S) 368 m cm^-1^.

### Refinement

Carbon-bound H-atoms were placed in calculated positions (C—H 0.95 to 0.99 Å) and were included in the refinement in the riding model approximation, with *U*_iso_(H) = 1.2 to 1.5*U*_equiv_(C). The oxygen-bound H-atom was refined with O—H = 0.84±0.01 Å and *U*_iso_(H) = 1.5*U*_equiv_(O).

### Crystal data

**Table:**

[Zn(C_6_H_12_NOS_2_)Cl(C_10_H_8_N_2_)]	*F*(000) = 896
*M**_r_* = 435.29	*D*_x_ = 1.588 Mg m^−^^3^
Monoclinic, *P*2_1_/*n*	Mo *K*α radiation, λ = 0.71073 Å
Hall symbol: -P 2yn	Cell parameters from 4897 reflections
*a* = 14.5008 (10) Å	θ = 2.4–28.7°
*b* = 8.6216 (4) Å	µ = 1.73 mm^−^^1^
*c* = 15.9905 (9) Å	*T* = 100 K
β = 114.423 (7)°	Block, colourless
*V* = 1820.25 (18) Å^3^	0.35 × 0.20 × 0.12 mm
*Z* = 4	

### Data collection

**Table:**

Oxford Diffraction Xcaliber Eos Gemini diffractometer	4061 independent reflections
Radiation source: fine-focus sealed tube	3603 reflections with *I* > 2σ(*I*)
Graphite monochromator	*R*_int_ = 0.034
Detector resolution: 16.1952 pixels mm^-1^	θ_max_ = 27.5°, θ_min_ = 2.5°
ω scans	*h* = −17→18
Absorption correction: multi-scan (*CrysAlis PRO*; Agilent, 2011)	*k* = −11→9
*T*_min_ = 0.882, *T*_max_ = 1.000	*l* = −20→20
11927 measured reflections	

### Refinement

**Table:**

Refinement on *F*^2^	Primary atom site location: structure-invariant direct methods
Least-squares matrix: full	Secondary atom site location: difference Fourier map
*R*[*F*^2^ > 2σ(*F*^2^)] = 0.031	Hydrogen site location: inferred from neighbouring sites
*wR*(*F*^2^) = 0.071	H atoms treated by a mixture of independent and constrained refinement
*S* = 1.04	*w* = 1/[σ^2^(*F*_o_^2^) + (0.0245*P*)^2^ + 1.5375*P*] where *P* = (*F*_o_^2^ + 2*F*_c_^2^)/3
4061 reflections	(Δ/σ)_max_ = 0.001
222 parameters	Δρ_max_ = 0.83 e Å^−^^3^
1 restraint	Δρ_min_ = −0.37 e Å^−^^3^

### Special details

**Table:**

Geometry. All s.u.'s (except the s.u. in the dihedral angle between two l.s. planes) are estimated using the full covariance matrix. The cell s.u.'s are taken into account individually in the estimation of s.u.'s in distances, angles and torsion angles; correlations between s.u.'s in cell parameters are only used when they are defined by crystal symmetry. An approximate (isotropic) treatment of cell s.u.'s is used for estimating s.u.'s involving l.s. planes.
Refinement. Refinement of *F*^2^ against ALL reflections. The weighted *R*-factor *wR* and goodness of fit *S* are based on *F*^2^, conventional *R*-factors *R* are based on *F*, with *F* set to zero for negative *F*^2^. The threshold expression of *F*^2^ > 2σ(*F*^2^) is used only for calculating *R*-factors(gt) *etc*. and is not relevant to the choice of reflections for refinement. *R*-factors based on *F*^2^ are statistically about twice as large as those based on *F*, and *R*- factors based on ALL data will be even larger.

### Fractional atomic coordinates and isotropic or equivalent isotropic displacement parameters (Å^2^)

**Table:**

	*x*	*y*	*z*	*U*_iso_*/*U*_eq_	
Zn	0.330691 (19)	0.17193 (3)	0.503343 (15)	0.01377 (8)	
Cl1	0.35450 (4)	0.06878 (6)	0.38450 (3)	0.01704 (12)	
S1	0.17619 (4)	0.07781 (6)	0.51151 (3)	0.01631 (12)	
S2	0.38248 (4)	−0.02253 (6)	0.63081 (3)	0.01617 (12)	
O1	0.29263 (14)	−0.1242 (2)	0.85472 (11)	0.0279 (4)	
H1o	0.294 (2)	−0.194 (2)	0.8922 (15)	0.030*	
N1	0.21773 (14)	−0.1346 (2)	0.64509 (12)	0.0164 (4)	
N2	0.44495 (14)	0.3303 (2)	0.58105 (11)	0.0142 (4)	
N3	0.26566 (14)	0.3969 (2)	0.45052 (11)	0.0148 (4)	
C1	0.25446 (16)	−0.0378 (2)	0.60191 (13)	0.0146 (4)	
C2	0.28503 (17)	−0.2471 (3)	0.71296 (14)	0.0183 (5)	
H2A	0.3303	−0.2945	0.6878	0.022*	
H2B	0.2429	−0.3310	0.7211	0.022*	
C3	0.34975 (19)	−0.1781 (3)	0.80665 (15)	0.0239 (5)	
H3A	0.3983	−0.2578	0.8444	0.029*	
H3B	0.3894	−0.0906	0.7984	0.029*	
C4	0.10593 (17)	−0.1546 (3)	0.61485 (15)	0.0179 (5)	
H4	0.0724	−0.0613	0.5774	0.021*	
C5	0.07640 (18)	−0.1600 (3)	0.69567 (15)	0.0225 (5)	
H5A	0.0974	−0.2595	0.7276	0.034*	
H5B	0.0028	−0.1489	0.6733	0.034*	
H5C	0.1099	−0.0752	0.7382	0.034*	
C6	0.06906 (18)	−0.2952 (3)	0.55214 (15)	0.0238 (5)	
H6A	0.0830	−0.2801	0.4977	0.036*	
H6B	−0.0040	−0.3080	0.5332	0.036*	
H6C	0.1045	−0.3882	0.5852	0.036*	
C7	0.53526 (17)	0.2870 (3)	0.64499 (14)	0.0181 (5)	
H7	0.5487	0.1796	0.6569	0.022*	
C8	0.61006 (17)	0.3927 (3)	0.69449 (15)	0.0198 (5)	
H8	0.6734	0.3585	0.7396	0.024*	
C9	0.59019 (17)	0.5490 (3)	0.67659 (14)	0.0198 (5)	
H9	0.6401	0.6241	0.7092	0.024*	
C10	0.49646 (17)	0.5954 (3)	0.61041 (14)	0.0172 (5)	
H10	0.4815	0.7024	0.5974	0.021*	
C11	0.42524 (16)	0.4830 (2)	0.56367 (13)	0.0138 (4)	
C12	0.32424 (16)	0.5203 (3)	0.48989 (13)	0.0141 (4)	
C13	0.29299 (18)	0.6707 (3)	0.46101 (14)	0.0188 (5)	
H13	0.3355	0.7563	0.4897	0.023*	
C14	0.19875 (19)	0.6939 (3)	0.38968 (14)	0.0215 (5)	
H14	0.1757	0.7960	0.3692	0.026*	
C15	0.13850 (18)	0.5670 (3)	0.34845 (15)	0.0224 (5)	
H15	0.0740	0.5801	0.2989	0.027*	
C16	0.17453 (17)	0.4209 (3)	0.38117 (14)	0.0199 (5)	
H16	0.1331	0.3336	0.3535	0.024*	

### Atomic displacement parameters (Å^2^)

**Table:**

	*U*^11^	*U*^22^	*U*^33^	*U*^12^	*U*^13^	*U*^23^
Zn	0.01493 (14)	0.01139 (14)	0.01590 (12)	−0.00257 (10)	0.00730 (10)	−0.00175 (9)
Cl1	0.0189 (3)	0.0167 (3)	0.0165 (2)	0.0009 (2)	0.0083 (2)	−0.00189 (19)
S1	0.0165 (3)	0.0150 (3)	0.0181 (2)	0.0020 (2)	0.0078 (2)	0.0045 (2)
S2	0.0143 (3)	0.0157 (3)	0.0171 (2)	−0.0017 (2)	0.0050 (2)	0.0012 (2)
O1	0.0363 (11)	0.0248 (10)	0.0236 (8)	−0.0009 (8)	0.0135 (8)	0.0025 (7)
N1	0.0153 (9)	0.0171 (10)	0.0164 (8)	0.0000 (8)	0.0063 (7)	0.0021 (7)
N2	0.0166 (9)	0.0127 (9)	0.0157 (8)	−0.0020 (7)	0.0091 (7)	−0.0006 (7)
N3	0.0163 (9)	0.0140 (9)	0.0156 (8)	−0.0029 (8)	0.0081 (7)	−0.0023 (7)
C1	0.0166 (11)	0.0131 (11)	0.0144 (9)	0.0006 (9)	0.0067 (8)	−0.0018 (8)
C2	0.0184 (11)	0.0154 (12)	0.0199 (10)	0.0021 (10)	0.0066 (9)	0.0059 (9)
C3	0.0245 (13)	0.0265 (13)	0.0193 (11)	−0.0029 (11)	0.0077 (9)	0.0032 (9)
C4	0.0144 (11)	0.0188 (12)	0.0209 (10)	−0.0009 (9)	0.0077 (9)	0.0033 (9)
C5	0.0186 (12)	0.0288 (14)	0.0226 (11)	−0.0058 (10)	0.0110 (9)	−0.0003 (10)
C6	0.0164 (12)	0.0291 (14)	0.0239 (11)	−0.0027 (11)	0.0064 (9)	−0.0041 (10)
C7	0.0169 (11)	0.0187 (12)	0.0209 (10)	0.0005 (10)	0.0099 (9)	0.0026 (9)
C8	0.0155 (11)	0.0261 (13)	0.0178 (10)	−0.0018 (10)	0.0070 (8)	0.0007 (9)
C9	0.0186 (12)	0.0250 (13)	0.0184 (10)	−0.0082 (10)	0.0103 (9)	−0.0058 (9)
C10	0.0197 (12)	0.0154 (11)	0.0191 (10)	−0.0030 (9)	0.0107 (9)	−0.0033 (8)
C11	0.0178 (11)	0.0126 (11)	0.0150 (9)	−0.0035 (9)	0.0105 (8)	−0.0020 (8)
C12	0.0187 (11)	0.0142 (11)	0.0125 (9)	−0.0011 (9)	0.0096 (8)	−0.0022 (8)
C13	0.0274 (13)	0.0127 (11)	0.0162 (10)	−0.0017 (10)	0.0089 (9)	−0.0008 (8)
C14	0.0298 (14)	0.0176 (12)	0.0171 (10)	0.0048 (10)	0.0096 (9)	0.0030 (9)
C15	0.0207 (12)	0.0267 (13)	0.0177 (10)	0.0032 (10)	0.0059 (9)	0.0003 (9)
C16	0.0179 (12)	0.0233 (13)	0.0176 (10)	−0.0029 (10)	0.0065 (9)	−0.0020 (9)

### Geometric parameters (Å, º)

**Table:**

Zn—Cl1	2.2503 (5)	C5—H5A	0.9800
Zn—S1	2.4366 (6)	C5—H5B	0.9800
Zn—S2	2.5026 (6)	C5—H5C	0.9800
Zn—N2	2.1097 (18)	C6—H6A	0.9800
Zn—N3	2.1692 (18)	C6—H6B	0.9800
S1—C1	1.736 (2)	C6—H6C	0.9800
S2—C1	1.723 (2)	C7—C8	1.386 (3)
O1—C3	1.421 (3)	C7—H7	0.9500
O1—H1o	0.844 (10)	C8—C9	1.383 (3)
N1—C1	1.327 (3)	C8—H8	0.9500
N1—C2	1.482 (3)	C9—C10	1.392 (3)
N1—C4	1.498 (3)	C9—H9	0.9500
N2—C7	1.339 (3)	C10—C11	1.388 (3)
N2—C11	1.351 (3)	C10—H10	0.9500
N3—C16	1.344 (3)	C11—C12	1.487 (3)
N3—C12	1.344 (3)	C12—C13	1.388 (3)
C2—C3	1.522 (3)	C13—C14	1.386 (3)
C2—H2A	0.9900	C13—H13	0.9500
C2—H2B	0.9900	C14—C15	1.386 (3)
C3—H3A	0.9900	C14—H14	0.9500
C3—H3B	0.9900	C15—C16	1.381 (3)
C4—C5	1.521 (3)	C15—H15	0.9500
C4—C6	1.523 (3)	C16—H16	0.9500
C4—H4	1.0000		
			
N2—Zn—N3	76.10 (7)	C4—C5—H5A	109.5
N2—Zn—Cl1	113.32 (5)	C4—C5—H5B	109.5
N3—Zn—Cl1	102.61 (5)	H5A—C5—H5B	109.5
N2—Zn—S1	134.39 (5)	C4—C5—H5C	109.5
N3—Zn—S1	93.28 (5)	H5A—C5—H5C	109.5
Cl1—Zn—S1	112.28 (2)	H5B—C5—H5C	109.5
N2—Zn—S2	93.20 (5)	C4—C6—H6A	109.5
N3—Zn—S2	148.41 (5)	C4—C6—H6B	109.5
Cl1—Zn—S2	108.90 (2)	H6A—C6—H6B	109.5
S1—Zn—S2	72.914 (19)	C4—C6—H6C	109.5
C1—S1—Zn	86.37 (8)	H6A—C6—H6C	109.5
C1—S2—Zn	84.58 (7)	H6B—C6—H6C	109.5
C3—O1—H1o	107 (2)	N2—C7—C8	122.7 (2)
C1—N1—C2	120.56 (19)	N2—C7—H7	118.6
C1—N1—C4	121.19 (18)	C8—C7—H7	118.6
C2—N1—C4	117.28 (17)	C9—C8—C7	118.4 (2)
C7—N2—C11	119.03 (19)	C9—C8—H8	120.8
C7—N2—Zn	123.48 (15)	C7—C8—H8	120.8
C11—N2—Zn	117.49 (14)	C8—C9—C10	119.5 (2)
C16—N3—C12	118.67 (19)	C8—C9—H9	120.3
C16—N3—Zn	125.42 (15)	C10—C9—H9	120.3
C12—N3—Zn	115.87 (14)	C11—C10—C9	118.9 (2)
N1—C1—S2	121.89 (16)	C11—C10—H10	120.5
N1—C1—S1	121.97 (17)	C9—C10—H10	120.5
S2—C1—S1	116.13 (12)	N2—C11—C10	121.5 (2)
N1—C2—C3	114.61 (19)	N2—C11—C12	115.40 (18)
N1—C2—H2A	108.6	C10—C11—C12	123.1 (2)
C3—C2—H2A	108.6	N3—C12—C13	121.8 (2)
N1—C2—H2B	108.6	N3—C12—C11	115.14 (19)
C3—C2—H2B	108.6	C13—C12—C11	123.0 (2)
H2A—C2—H2B	107.6	C14—C13—C12	119.0 (2)
O1—C3—C2	113.6 (2)	C14—C13—H13	120.5
O1—C3—H3A	108.8	C12—C13—H13	120.5
C2—C3—H3A	108.8	C13—C14—C15	119.4 (2)
O1—C3—H3B	108.8	C13—C14—H14	120.3
C2—C3—H3B	108.8	C15—C14—H14	120.3
H3A—C3—H3B	107.7	C16—C15—C14	118.4 (2)
N1—C4—C5	112.13 (17)	C16—C15—H15	120.8
N1—C4—C6	110.02 (18)	C14—C15—H15	120.8
C5—C4—C6	112.90 (19)	N3—C16—C15	122.8 (2)
N1—C4—H4	107.2	N3—C16—H16	118.6
C5—C4—H4	107.2	C15—C16—H16	118.6
C6—C4—H4	107.2		
			
N2—Zn—S1—C1	−77.16 (10)	C4—N1—C2—C3	−113.0 (2)
N3—Zn—S1—C1	−150.87 (8)	N1—C2—C3—O1	66.0 (3)
Cl1—Zn—S1—C1	104.07 (7)	C1—N1—C4—C5	−137.2 (2)
S2—Zn—S1—C1	0.17 (7)	C2—N1—C4—C5	54.0 (3)
N2—Zn—S2—C1	135.54 (9)	C1—N1—C4—C6	96.3 (2)
N3—Zn—S2—C1	67.16 (12)	C2—N1—C4—C6	−72.5 (2)
Cl1—Zn—S2—C1	−108.48 (7)	C11—N2—C7—C8	0.0 (3)
S1—Zn—S2—C1	−0.17 (7)	Zn—N2—C7—C8	179.02 (16)
N3—Zn—N2—C7	−178.42 (18)	N2—C7—C8—C9	−0.2 (3)
Cl1—Zn—N2—C7	−80.48 (17)	C7—C8—C9—C10	0.3 (3)
S1—Zn—N2—C7	100.76 (17)	C8—C9—C10—C11	−0.2 (3)
S2—Zn—N2—C7	31.69 (16)	C7—N2—C11—C10	0.1 (3)
N3—Zn—N2—C11	0.66 (14)	Zn—N2—C11—C10	−179.00 (15)
Cl1—Zn—N2—C11	98.60 (15)	C7—N2—C11—C12	178.38 (18)
S1—Zn—N2—C11	−80.16 (16)	Zn—N2—C11—C12	−0.7 (2)
S2—Zn—N2—C11	−149.23 (14)	C9—C10—C11—N2	0.0 (3)
N2—Zn—N3—C16	177.47 (18)	C9—C10—C11—C12	−178.10 (19)
Cl1—Zn—N3—C16	66.22 (17)	C16—N3—C12—C13	0.2 (3)
S1—Zn—N3—C16	−47.48 (17)	Zn—N3—C12—C13	178.27 (16)
S2—Zn—N3—C16	−109.55 (17)	C16—N3—C12—C11	−177.85 (18)
N2—Zn—N3—C12	−0.47 (14)	Zn—N3—C12—C11	0.2 (2)
Cl1—Zn—N3—C12	−111.72 (14)	N2—C11—C12—N3	0.3 (3)
S1—Zn—N3—C12	134.57 (14)	C10—C11—C12—N3	178.54 (19)
S2—Zn—N3—C12	72.51 (18)	N2—C11—C12—C13	−177.68 (19)
C2—N1—C1—S2	−7.2 (3)	C10—C11—C12—C13	0.5 (3)
C4—N1—C1—S2	−175.69 (15)	N3—C12—C13—C14	0.1 (3)
C2—N1—C1—S1	171.42 (15)	C11—C12—C13—C14	177.9 (2)
C4—N1—C1—S1	2.9 (3)	C12—C13—C14—C15	−0.6 (3)
Zn—S2—C1—N1	178.96 (18)	C13—C14—C15—C16	0.8 (3)
Zn—S2—C1—S1	0.26 (11)	C12—N3—C16—C15	0.1 (3)
Zn—S1—C1—N1	−178.97 (18)	Zn—N3—C16—C15	−177.77 (17)
Zn—S1—C1—S2	−0.26 (11)	C14—C15—C16—N3	−0.6 (3)
C1—N1—C2—C3	78.0 (3)		

### Hydrogen-bond geometry (Å, º)

**Table:**

*D*—H···*A*	*D*—H	H···*A*	*D*···*A*	*D*—H···*A*
O1—H1*o*···S1^i^	0.84 (2)	2.42 (2)	3.2528 (18)	167 (2)
C6—H6c···O1^i^	0.98	2.47	3.438 (3)	169
C13—H13···S2^ii^	0.95	2.81	3.625 (2)	144
C7—H7···Cl1^iii^	0.95	2.79	3.579 (3)	142
C8—H8···Cl1^iv^	0.95	2.76	3.606 (2)	148

Symmetry codes: (i) −*x*+1/2, *y*−1/2, −*z*+3/2; (ii) *x*, *y*+1, *z*; (iii) −*x*+1, −*y*, −*z*+1; (iv) *x*+1/2, −*y*+1/2, *z*+1/2.
